# Predicting cortical bone adaptation to axial loading in the mouse tibia

**DOI:** 10.1098/rsif.2015.0590

**Published:** 2015-09-06

**Authors:** A. F. Pereira, B. Javaheri, A. A. Pitsillides, S. J. Shefelbine

**Affiliations:** 1 Department of Bioengineering, Imperial College London, London, UK; 2 Comparative Biomedical Sciences, Royal Veterinary College, London, UK; 3 Department of Mechanical and Industrial Engineering and Department of Bioengineering, Northeastern University, Boston, MA, USA

**Keywords:** bone mechanobiology, functional adaptation, fluid flow, cortical thickness, mouse tibia

## Abstract

The development of predictive mathematical models can contribute to a deeper understanding of the specific stages of bone mechanobiology and the process by which bone adapts to mechanical forces. The objective of this work was to predict, with spatial accuracy, cortical bone adaptation to mechanical load, in order to better understand the mechanical cues that might be driving adaptation. The axial tibial loading model was used to trigger cortical bone adaptation in C57BL/6 mice and provide relevant biological and biomechanical information. A method for mapping cortical thickness in the mouse tibia diaphysis was developed, allowing for a thorough spatial description of where bone adaptation occurs. Poroelastic finite-element (FE) models were used to determine the structural response of the tibia upon axial loading and interstitial fluid velocity as the mechanical stimulus. FE models were coupled with mechanobiological governing equations, which accounted for non-static loads and assumed that bone responds instantly to local mechanical cues in an on–off manner. The presented formulation was able to simulate the areas of adaptation and accurately reproduce the distributions of cortical thickening observed in the experimental data with a statistically significant positive correlation (Kendall's *τ* rank coefficient *τ* = 0.51, *p* < 0.001). This work demonstrates that computational models can spatially predict cortical bone mechanoadaptation to a time variant stimulus. Such models could be used in the design of more efficient loading protocols and drug therapies that target the relevant physiological mechanisms.

## 1. Introduction

Bone is a dynamic tissue, responding to changes in mechanical demands by adapting its shape and material properties. These self-optimizing properties derive from the formation of new bone under mechanical loads and loss of bone in disuse, a mechanism referred to as Wolff's law [1]. Classic examples of the adaptive effect of skeletal loading in humans include bone loss during exposure to microgravity environments [2] or bed rest confinement [3], and bone mass gain resulting from frequent physical exercise [4].

The mechanostat hypothesis by Frost [1] relies upon a quantitatively matched adaptive response of bone as a function of loading, considering that there are mechanical stimulus thresholds at which bone tissue responds. This conceptual model delineates the foundations of most of the mathematical models developed to simulate bone adaptation [5–13]. Such predictive mathematical models usually rely on numerical methods, namely finite-element analysis (FEA), to calculate mechanical fields in bone that result from external loading. These help to find correlations between the mechanical environment and biological response.

Several mechanical stimuli have been suggested to predict spatial patterns of adaptation of bone tissue, such as strain tensor [7], daily stress stimulus [5,6] or strain energy density [10]. Following findings suggesting that load-induced fluid flow is the likely mechanism for mechanocoupling [14–16], other authors have introduced Darcy fluid velocity as the mechanical stimulus. Such studies modelled pressure relaxation occurring in the lacunar–canalicular system during loading and showed that it can explain not only space-dependent but also time-dependent phenomena associated with some load parameters. These were able to take into account the effect of load frequency [17–19] or the insertion of rest periods between load cycles [20,21]. Understanding how loading parameters can be tuned to maximize the adaptive response will contribute to the development of new non-pharmacological interventions to stimulate bone formation.

To date, few studies have thoroughly examined the spatial arrangement of adapted cortical bone across the entire bone in relation to the mechanical field. In addition, simulations of cortical bone modelling are often tested against experimental data with the representation of a few cross sections, with the calculation of bulk quantities, such as minimum moment of area [17] or remodelling rate [19]. These approaches lack a thorough spatial representation of *in vivo* and simulated cortical adaptation. Roberts *et al*. [22] correlated site-specific distribution of new bone apposition with mechanical signals in an animal study on the synergistic effect of parathyroid hormone and localized mechanical stress; however, this analysis was limited to single cross sections. An approach where this assessment is done with higher spatial resolution is, therefore, required to fully assess the predictive ability of phenomenological models.

We hypothesize that bone forms in regions of high fluid velocity. The objective of this work was to predict, with spatial accuracy, cortical bone adaptation to mechanical load using fluid flow as the mechano-stimulus, in order to better understand the mechanical cues that might be driving adaptation. We conducted complementary *in vivo* and *in silico* studies. *In vivo* bone formation data were obtained using a murine tibial loading model [23]. Finite-element (FE) models that simulated the structural response of the loaded mouse tibia were developed and poroelastic theory was considered to estimate load-induced interstitial fluid velocities. These models were coupled to a novel mechanobiological formulation that predicts functional cortical bone thickening based on fluid velocity. We calculated changes in cortical thickness resulting from bone formation in real and simulated adaptation, in order to assess the accuracy of the predictive model.

## 2. Material and methods

### 2.1. Axial loading of the mouse tibia

Six (*N* = 6) 12-week-old female C57BL/6 J mice, kept in polypropylene cages, were subjected to 12 L : 12 D cycles of exposure.

**Figure 1.**
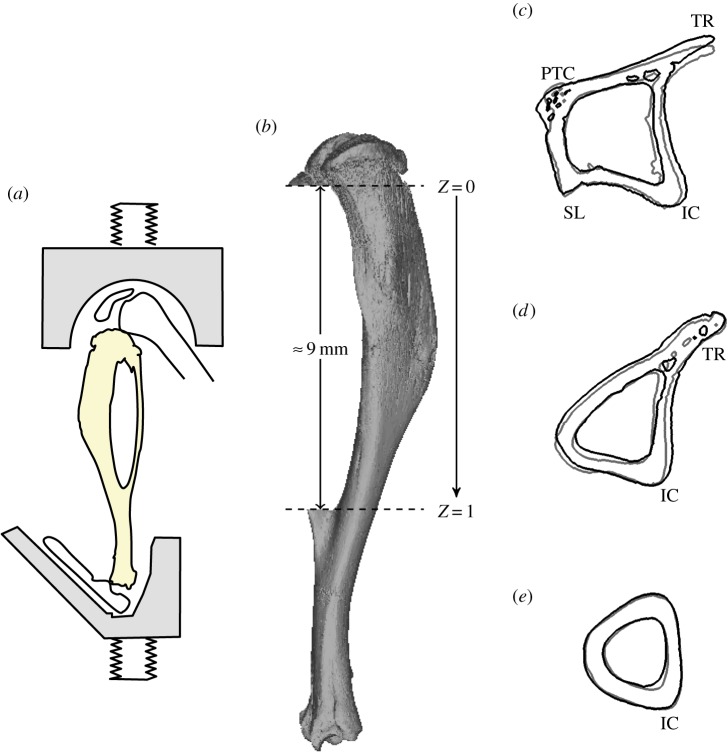
(*a*) The loading model used for this study: axial compression of the murine tibia (adapted from [23]). (*b*) Non-dimensional coordinate, *Z*, corresponds to the axial space between the proximal (*Z* = 0) and distal (*Z* = 1) tibia–fibula junctions. (*c*–*e*) Cross-sectional contours of left (non-loaded, grey line) and right (loaded, black line) tibiae for a particular specimen. Anatomical landmarks: interosseous crest (IC), proximal tibial crest (PTC), soleal line (SL), tibial ridge (TR). (*c*) *Z* = 0.2, (*d*) *Z* = 0.5 and (*e*) *Z* = 0.8.

A non-invasive bone adaptation model in mice was used for this experiment, identical to the one described in the work by De Souza *et al*. [23] and shown in figure 1*a*. The extrinsic actuator consisted of two vertically aligned cups. The cups were enclosed in a servo-hydraulic materials testing machine (model HC10; Dartec, Ltd, Stourbridge, UK), with an actuator and a load cell. The right tibia of each specimen was placed in between the cups, while the left limb served as a contralateral non-loaded control.

The natural curvature of the tibia induces bending and transforms the axial loading into a combination of compression and mediolateral bending. This loading model provides measurable amounts of bone formation after two weeks, avoids artefacts that arise from the effects of surgical procedures or skin pressure-induced periosteal adaptation (observable in flexural loading protocols [24]), and allows for the examination of periosteal, endosteal and trabecular adaptation [23].

Load cycles consisted in a 0.1 s trapezoidal load, in which peak loads of 13 N were applied for 0.05 s, with loading and unloading times of 0.025 s, separated by a 9.9 s baseline rest period [23,25]. These were applied at 40 cycles/day, three times a week for two weeks. Previous studies have shown that loads of 9–11 N elicit lamellar bone adaptation [23,26].

Tibiae of both left and right legs were carefully dissected, fixed in 70% EtOH and stored until scanning. We used the Skyscan 1172 micro-CT system (Skyscan, Kontich, Belgium) with the X-ray tube operated at 50 kV and 200 µA, 1600 ms exposure time with a 0.5 mm aluminium filter and a focal spot size of 5 µm. The slices were then reconstructed using NRecon 1.6.9.4 (Skyscan, Kontich, Belgium). The micro-CT transverse images were imported to an image-processing software (Mimics v. 15.1; Materialise, Leuven, Belgium), where the tibia was segmented from other regions containing pixels belonging to the femur and pes bones. STL meshes of right tibiae were reflected and registered to the corresponding contralateral bone using the Point Registration option in Mimics.

Both geometries were then aligned to the longitudinal axis, parallel to the line that passes through the tibia–fibula proximal and distal junction points. Dimension *Z* was considered to have the same direction and to be the axial range between the tibia–fibula proximal and distal junctions, in the distal direction (*Z* = 0 at the proximal junction and *Z* = 1 distally), as illustrated in figure 1*b*. Cross-sectional images, perpendicular to the *Z*-axis, were exported as BMP files and used to calculate the cortical thickness, as described below. Figure 1*c–e* shows the tibial cross sections of loaded (black line) and contralateral (grey line) limbs for an example specimen.

Four major anatomical landmarks are present in the diaphysis of the mouse tibia: the interosseous crest (labelled in figure 1*c*–*e* as IC), the tibial ridge (TR), the soleal line (SL) and the proximal crest (PC) [27]. The left limb tibia (non-loaded leg) was used in the FE simulations in §2.4.

### 2.2. Determining changes in cortical thickness from *in vivo* experiments

**Figure 2.**
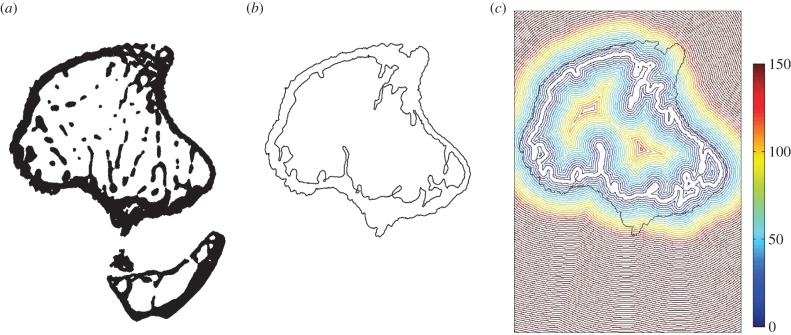
Steps of script for the calculation of cortical thickness and endosteal distance from the centroid. (*a*) Initial cross section, (*b*) peri- and endosteal boundaries and (*c*) map of distances from the endosteum (units in micrometres).

Transverse images of the bone (figure 2*a*) and Matlab (Mathworks, Natick, MA, USA) scripts were used to obtain a map of cortical thickness changes, from contralateral to loaded leg, that occur in the diaphysis of the tibia only, without considering the fibula. The automatic process started by segmenting tibial cortical bone from the fibula and unconnected trabecular structures. The exterior boundaries were identified and other features resulting from blood vessel and distinct porosities were removed (figure 2*b*). The thickness of cortical bone, in this study denoted as Th, was determined at each pixel in the periosteal boundary as the shortest distance to the endosteal boundary (figure 2*c*).

**Figure 3.**
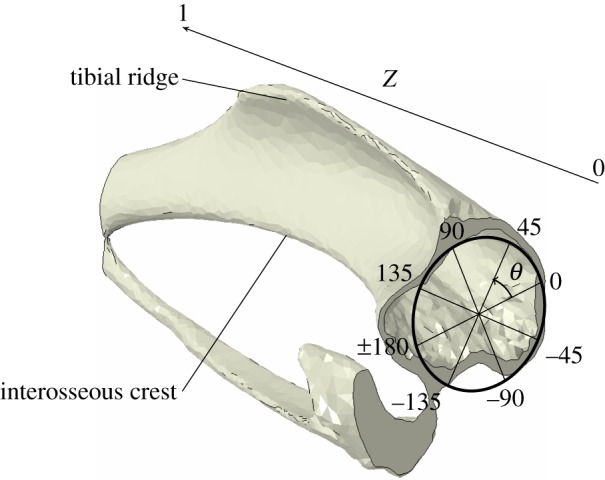
Angular (*θ*) and longitudinal (*Z*) coordinates used in the cylindrical coordinates system.

Local thickness measurements were arranged into groups according to their angular positions *θ* from the centroid of the cross section. For each group, the measured thicknesses were averaged for the pixels enclosed within an azimuthal range of *π*/90 (2°). Left and right leg results were expressed in a cylindrical coordinate system (Th, *θ*, *Z*), illustrated in figure 3, where the coordinates, respectively, correspond to the thickness of the cortical shell, the angular position in the cross section with the cortical centroid as origin and the longitudinal distance between the tibia–fibula proximal and distal junctions. The variation of cortical thickness shell (ΔTh/Th) between adapted and non-adapted tibiae, at angle *θ* and longitudinal position *Z*, is given by2.1
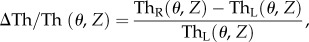
with Th_L_ and Th_R_ as the thickness values of the left and right legs, respectively.

Different animals had slight differences in the longitudinal and angular position of the tibial borders and other bony landmarks. However, these were enough to make statistical analysis of experimental data results rather challenging, concealing the desired adaptation patterns. We measured changes in the second moment of area in tibial cortical bone for all samples (electronic supplementary material, figure S1)^1^ and identified the one with the smallest least-squared error distance from the average. We assumed this sample to be the best representation of average adaptation seen in our experiments. We measured cortical thickness, Th, in the representative sample and its unloaded limb geometry was used as the initial state in the adaptation model.

### 2.3. Proposed mechanobiological model

**Figure 4.**
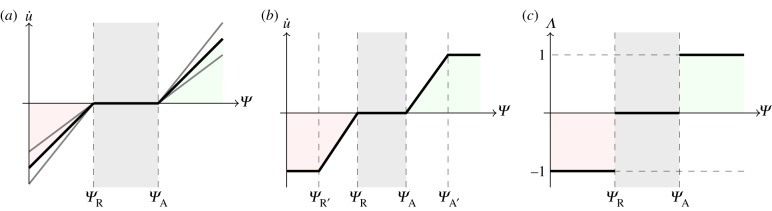
Different 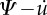 (mechanical stimulus versus adaptation) relationship curves considered in this study: (*a*) generic trilinear curve, (*b*) trilinear curve capped with maximum remodelling rates of apposition and resorption, (*c*) on–off relationship. The apposition and resorption limits of the homeostatic interval, *Ψ*_A_ and *Ψ*_R_, dictate the tissue response to mechanical stimulus *Ψ*. The step curve in (*c*) obtained the most accurate predictions of cortical adaptation.

An organ-scale predictive model of bone adaptation was developed to include non-static loading conditions, while considering load-induced interstitial fluid velocity as the mechanical signal. Adaptation of bone to a mechanical stimulus was determined with a trilinear curve, with high and low mechanical signals inducing, respectively, formation and resorption of bone, and a ‘lazy zone’ where bone formation and resorption are balanced (figure 4; [6,7,10]).

Coupling time integration (or time averaging) of the mechanical signal with previously published stimulus–adaptation relationships [17–19] resulted in excessive qualitative differences in the amount of surface modelling between regions of high and low signal, which were not seen in experimental results. We adjusted the slopes of the remodelling curve (figure 4*a*) and imposed maximum and minimum remodelling rates (figure 4*b*) to obtain regions of bone formation that correlated with experimental results. The closest predictions, in terms of spatial locations across the entire tibial shaft and relative amounts of adaptation, when compared with experimental results, were obtained with a step function profile (figure 4*c*). Therefore, our model assumes that, at specific time instances, bone responds to local mechanical cues in an on–off manner and that this response is integrated in time.

**Figure 5.**
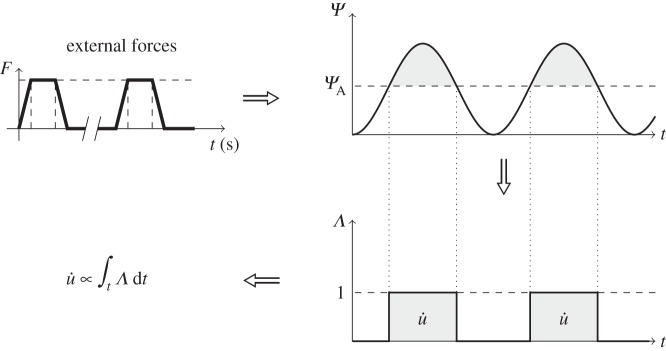
Cortical bone adaptation model. An externally applied force elicits a mechanical signal/stimulus, *Ψ*, in the bone. If this stimulus is above a certain apposition threshold, *Ψ*_A_, then an adaptive response is generated (*Λ* = 1). This response is integrated over time to give the adaptation rate (*u̇*).

The course of action of the presented adaptation model, schematized in figure 5 for a case where only bone formation is considered, is as follows. For the duration of a load cycle, non-static loading conditions result in varying mechanical signal *Ψ* values in time. The stimulus *Ψ* is converted into a discrete value, denoted as *Λ*, that corresponds to the cellular sensorial output to the mechanical signal. The adaptation rate, *u̇*, is obtained from the time integration of the cellular signal, finally resulting in bone surface modelling.

The mechanosensing signal calculated at node *n* and time instant *t*, *Ψ*(*n*, *t*), can elicit three discrete responses of bone activity, *Λ*(*n*, *t*), dictated by the relative value of the signal compared with the apposition and resorption limits of the homeostatic interval, *Ψ*_A_ and *Ψ*_R_. The value of *Λ* is a dimensionless quantity and can be expressed as the following set of equations:2.2
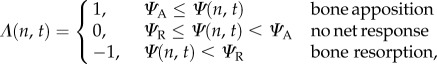
where *Λ* takes positive values at time periods where the mechanical signal is above the apposition set point *Ψ*_A_. The corresponding local remodelling rate *u̇* (units of length per iteration) is linearly proportional to the integral of *Λ* over time2.3
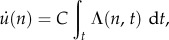
where *C* is a proportionality constant (units of length per iteration). This means that the remodelling rate will be higher the longer the signal stays above the threshold.

The arrangement of the osteocyte network suggests that mechanotransductive signals are transmitted spatially. This communication was modelled similar to Chennimalai Kumar *et al*. [17] by averaging the local adaptive response, *u̇*(*n*), over a spherical zone of influence of radius *R*. Averaging was weighted with function *w*(*r*) [28], which linearly decays with distance *r* from the node where adaptation is being computed2.4
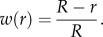


The value of *R* was set to be 150 µm, the average osteon radius [29], as it was assumed that the latter is associated with the physical limits of nutrient perfusion and osteocyte action reach.^2^

The averaging at a node *n*, *u̇*(*n*), was calculated as the mean between the value of *u̇*(*n*) and its weighted average at the other nodes inside the zone of influence2.5
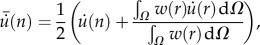


with *Ω* as a spherical region of integration, centred in *n* and excluding its centre.

The vector of nodal displacement rate 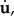 with the same direction as the node normal (as bone formation occurs by appositional growth), constitutes the final adaptive quantity to be calculated at the end of each remodelling iteration,2.6
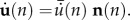


### 2.4. Adaptation algorithm and finite-element model

Poroelastic theory was used to model the biphasic nature of bone and predict fluid motion inside the lacunar canalicular pores [31]. FE modelling was employed to provide a numerical solution to the poroelastic problem.

**Figure 6.**
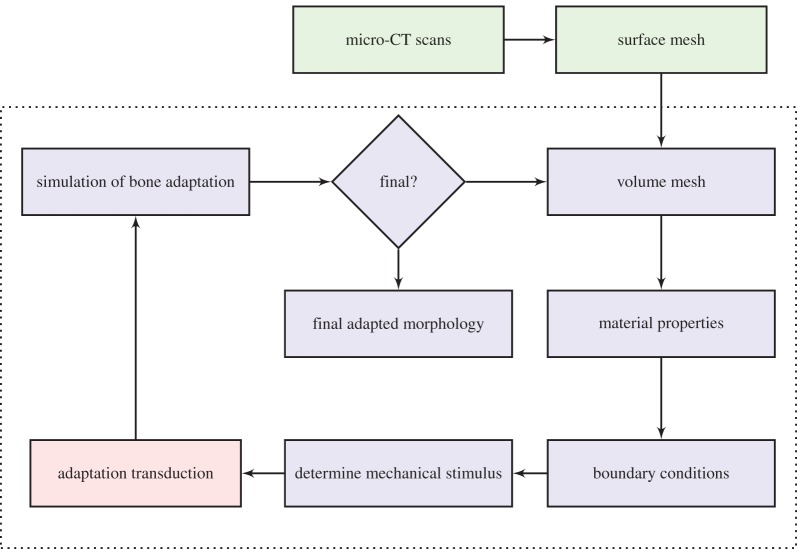
Adaptation algorithm flowchart. Shade indicative of software used: Mimics (green), ABAQUS (blue) and Matlab (red). Steps inside dashed line are automated.

Simulations of cortical bone adaptation (figure 6) iteratively executed the following steps: (1) generate an FE mesh of the unloaded limb, (2) determine the relevant mechanical fields experienced by cortical bone during experimental loading conditions (in this study, fluid velocity is considered the mechanical stimulus, *Ψ* = *V*), (3) apply the adaptation model described in §2.3, and (4) update the geometry of bone accordingly.

The solver was run in the High Performance Computing facility at Imperial College London, UK. FE model generation and handling (steps 1 and 4) were implemented using the Abaqus (v. 6.12; ABAQUS Inc., Pawtucket, RI, USA) Python-based scripting extension. Matlab was used to post-process the FE results and calculate the remodelling rate (step 3).

Only 0.5 s of total loading time was processed, in order to have feasible computation times, enclosing a single load cycle (0.1 s) and allowing pressure relaxation to take place in the remaining 0.4 s. The solver time step was set to 0.005 s, in order to account for the load cycle profile, totalling 100 time frames per loading cycle.

#### 2.4.1. Material properties

**Figure 7.**
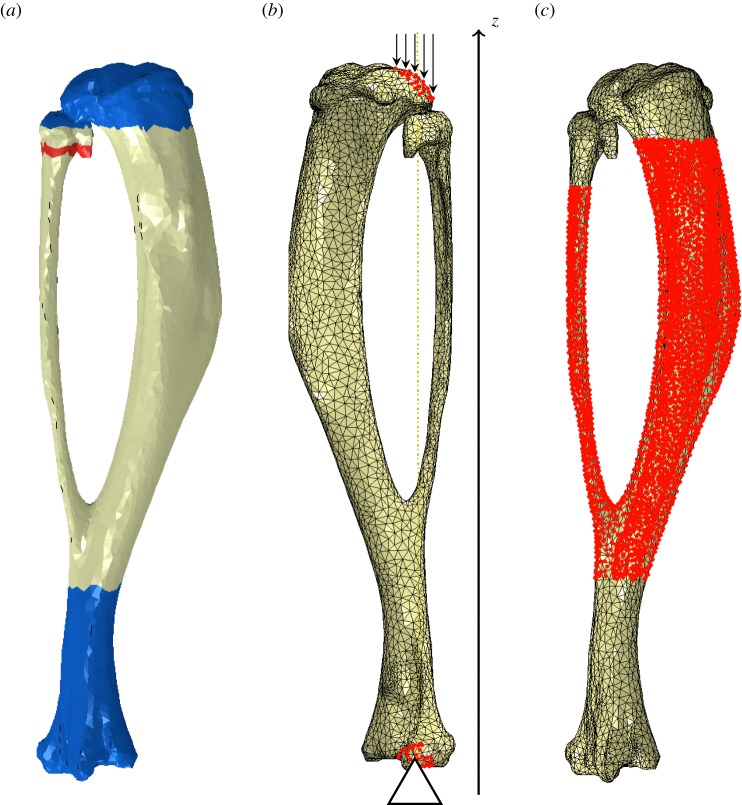
FE model used to determine the mechanical environment. (*a*) Medial view of the material sections considered: poroelastic cortical bone (off-white), elastic cortical bone (blue) and fibular growth plate (red). (*b*) Lateral view of the boundary conditions applied and vertical alignment of the model assembly with the *z*-direction. (*c*) Medial view of the region where cortical bone adaptation was considered, demarcated by the cloud of nodes highlighted in red.

The mouse tibia was modelled using four material sections, shown in figure 7*a*; elastic and poroelastic cortical bone, fibular growth plate and membrane layers (not shown). Cortical bone elements were modelled as transversely isotropic, linear elastic and homogeneous [32]. Elements within the fibular growth plate were assumed to be cartilage, modelled as isotropic [33].^3^ Predicted surface strains were compared against experimental data, obtained in a previous study by Sztefek *et al*. [34], acquired using digital image correlation (DIC) and strain gauges, in eight-week-old mice of the same strain and gender.

The poroelastic region (tibial diaphysis in figure 7*a*) was defined as the elements enclosed within and surrounding the adaptive region. Elastic elements were considered in the rest of the cortical region (blue colour in figure 7*a*), where no adaptation was considered. Intrinsic permeability in the poroelastic bone regions was set to *k* = 10^−22^ m^2^ [20]. Osteocyte canaliculi are predominantly oriented longitudinally, rather than transversely [35], and, therefore, this value was assigned to the longitudinal component, *k*_1_. Permeability in other directions was assumed to be 10 times smaller. We modelled permeability through the periosteal and endosteal membranes by including a layer of poroelastic elements, similar to Steck *et al*. [32], and considered each surface to have an isotropic permeability of *k* = 10^−17^ m^2^ [36]. The arrangement of membrane layers is not representative, as a much larger thickness was required to obtain good quality elements; however, this membrane thickness did not affect the mechanical stimulus in the bone, *Ψ*.

The material properties used for bone, fibular growth plate and membrane layers sections are listed in table 1. The properties for the interstitial fluid, listed in table 2, were assumed to be similar to saline. Some of the constitutive values that were used were estimated or measured for different specimens, as the literature lacks estimation of some poroelastic parameters in murine bone.

**Table 1.** Material properties used for cortical bone, membrane layers and growth plate elements.

property		units	bone	membrane layers	growth plate elements
longitudinal Young's modulus	*E*_z_	MPa	17.0 × 10^3^ [32]	2 [33]	10.0 [33]
transverse Young's modulus	*E*_t_	MPa	11.5 × 10^3^ [32]	2 [33]	10.0 [33]
transverse Poisson's ratio	*v*_t_	—	0.38 [32]	0.167 [33]	0.167 [33]
longitudinal–transverse Poisson's ratio	*v*_lt_	—	0.41 [32]	0.167 [33]	0.167 [33]
transverse shear modulus	*G*_t_	MPa	4.1 × 10^3^ [32]	0.73 [33]	6.86 [33]
longitudinal–transverse shear modulus	*G*_It_	MPa	5.2 × 10^3^ [32]	0.73 [33]	6.86 [33]
pore volume fraction	*ϕ*	%	5 [31]	0.8 [33]	—
longitudinal permeability	*k*_l_	m^2^	1 × 10^−22^ [20]	1 × 10^−17^ [36]	—
transverse permeability	*k*_t_	m^2^	1 × 10^−23^ [20,35]	1 × 10^−17^ [36]	—
effective stress coefficient	*α*	—	0.14 [31]	—	—

**Table 2.** Interstitial fluid material properties (adapted from [31]).

property		units	fluid
specific weight	*γ*	N m^–3^	9.8 × 10^3^
dynamic viscosity	*μ*	Pa s	8.9 × 10^−4^
bulk modulus	*K_f_*	MPa	2.3 × 10^3^

Sensitivity studies (not included in this paper) were conducted to determine the influence of trabecular bone and growth plate stiffness on the calculations of the mechanical environment in the diaphysis. A wide interval of stiffness values was considered for each compartment, ranging from cartilage (10 MPa) to cortical bone (17 GPa). Estimations of *Ψ* were insensitive to the properties of the tibial growth plate (which is present even in skeletally mature mice), epiphyseal and metaphyseal trabecular bone. Hence, the growth plate and epiphysis were modelled as solid bone (*E* = 17 GPa) and the metaphyseal trabecular compartment was modelled as hollow. The mechanical fields obtained in the fibula were, however, influenced greatly by the material properties of the fibular growth plate, which was modelled as a cartilage layer (≈3.5 µm thick with modulus *E* = 10 MPa).

#### 2.4.2. Finite-element mesh

Using the segmented images from §2.1, the shrink-wrap meshing tool in Mimics was employed to close small openings and apply smoothing to the geometry, set with a smallest detail of 50 µm and a gap closing distance of 200 µm, followed by a triangle reduction with a tolerance of 10 µm and an edge angle of 30°. The obtained surface mesh was then remeshed using the Height/Base(N) shape measurement, a maximum geometric error of 10 µm and a maximum triangle edge length of 30 µm. The final surface mesh, consisting on two shells (representing the endosteal and periosteal surfaces), was exported and used as the starting template for our adaptation algorithm.

Surface normal vectors at each node *n*, **n**(*n*), were calculated as the normalized average of the normals of the triangular elements that contain node *n*. Normal directions for periosteal and endosteal boundaries were defined, respectively, as the outer-pointing and the inward-pointing.

Elastic and poroelastic regions, shown in figure 7*a*, were respectively meshed with C3D10 (3D, 10-node tetrahedral quadratic) and C3D10MP (with a pore pressure degree of freedom) elements. The volume was remeshed at the start of each iteration of the adaptation algorithm, maintaining the same surface nodes and prolonging mesh quality. The poroelastic section of the initial volume mesh had a distance between closest nodes between 13 and 112 µm, with an average distance of 47.39 µm.

#### 2.4.3. Boundary conditions

Loading conditions were determined from a study by Poulet *et al*. [25], where micro-CT imaging was employed to visualize the knee joint during axial loading. Axial loading described in §2.1 was modelled by using concentrated forces distributed by a node set at the proximal condyles and fixing set of nodes at the distal interior articular surface (figure 7*b*). The loads resulted in a total of 13 N applied as a single trapezoidal load cycle, with a load and unload time of 0.025 s and a peak load time of 0.05 s, in line with the *in vivo* studies. At each iteration, the direction of the applied forces was vertically aligned with the distal fixed nodes region, in order to avoid unrealistic bending moments.

Layers of elements adjacent to both the outer and inner surfaces modelled the flow boundary conditions at the periosteal and endosteal membranes [32]. By default, the regions adjacent to the outer surface of the layers had no fluid pressure gradients and, therefore, a zero flow condition was imposed in these boundaries.

#### 2.4.4. Mechanobiological parameters used

In order to determine the influence of the reference stimulus, *Ψ*_A_, and adjust this parameter to fit the experimental data, five different values for the stimulus threshold were considered, as listed in table 3. An arbitrary value was used for *C* (4.45 µm iteration^−1^); a value large enough to have substantial bone formation between consecutive adaptive iterations, yet small enough to avoid over-deformation of the mesh and of the same order of magnitude as the maximum daily mineral apposition rate calculated from the micro-CT scans (≈6.68 µm d^−1^). In this pro-osteogenic model, we did not consider resorption (*Ψ*_R_ = 0). Table 4 shows the rest of the parameters employed in the simulation.

**Table 3.** Values used for the bone apposition reference stimulus, *Ψ*_A_.

	1	2	3	4	5
*Ψ*_A_ (µm s^–1^)	1.0 × 10^−3^	3.0 × 10^−2^	6.0 × 10^−2^	7.0 × 10^−2^	1.0 × 10^−1^

**Table 4.** Values used for the other parameters of the adaptation model.

property	*C* (µm/interation)	*Ψ*_R_ (MPa)	*R* (µm)
value	4.45	0	150

Surface nodes, where adaptation was considered (figure 7*c*), had at least 10 nodes within the spherical zone of influence with radius *R* = 150 µm, and, in denser mesh sections, up to 212 nodes were contained. The whole set of surface nodes had an average of 45.32 neighbour nodes.

#### 2.4.5. Changes in morphology

The adaptive region was restricted to the tibial diaphysis, as shown in figure 7*c*. Mechanical signals were rendered into surface node displacements, in a direction normal to the bone's surface, simulating bone apposition. Geometry changes were accomplished by translocating surface nodes followed by the generation of a new volume mesh. Changes in bone shape were assessed by measuring cortical thickening of cross-sectional cuts of the resulting mesh morphology, obtained at each iteration. We determined the maximum change in thickness in the adapting region from the experimental data. The maximum change in thickness for the bone modelled was ΔTh/Th ≈ 0.67. We stopped iterations when a region in our simulations reached the experimental maximum thickness value.

To assess the accuracy of the cortical adaptation predictions, we used Matlab to compute the correlation between experimental and computational ΔTh/Th measurements, for each value of *Ψ*_A_. The changes in thickness calculations, presented in the following sections, deviate significantly from normal distribution, as tested by the Kolmogorov–Smirnov test (*p* < 0.001). Therefore, we used Kendall's *τ* rank correlation coefficient, a non-parametric correlation test, to compare experimental measurements with the results of our predictive model.

## 3. Results

### 3.1. Digital image correlation verification of surface strains

**Figure 8.**
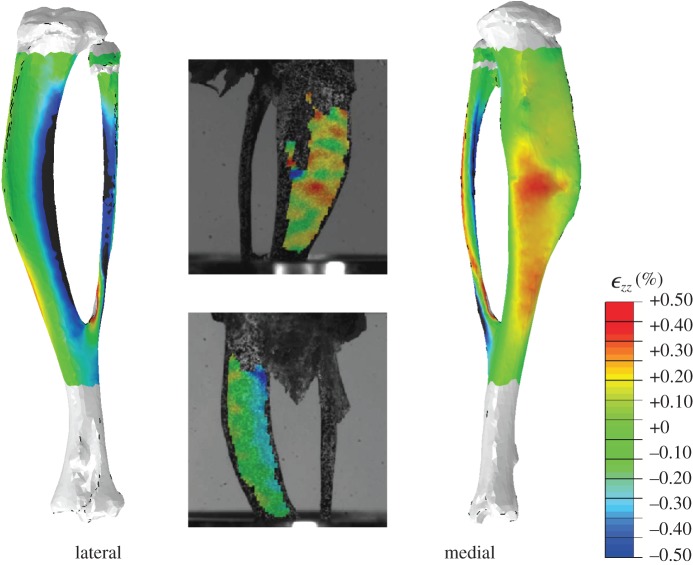
Contour plots of the longitudinal strains 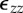 calculated in the FE models and DIC measurements in eight-week-old mice of the same strain and gender by Sztefek *et al*. [34]. FEA used the geometry of a non-adapted tibia under an axial peak load of 12 N. (DIC images adapted from [34] with permission from Elsevier.)

Figure 8 shows the contour plots of the longitudinal strains 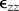 calculated in the FE predictions against the DIC measurements by Sztefek *et al*. [34], for a non-adapted left tibia. The DIC readings were obtained for a 12 N axial load (the experimental protocol applied 13 N) and thus the force in the FE model was adjusted accordingly, for this verification step.

These plots show inhomogeneous regions of high strain, with tension on the medial surface and compression on the lateral side, specifically on the interosseous crest. Qualitatively, FE strain patterns agree well with the experimental measurements, with a slight overestimation in the peak compressive strains. These results encouraged the use of these FE models to estimate the mechanical stimulus for adaptation.

### 3.2. Mechanical stimulus calculations

**Figure 9.**
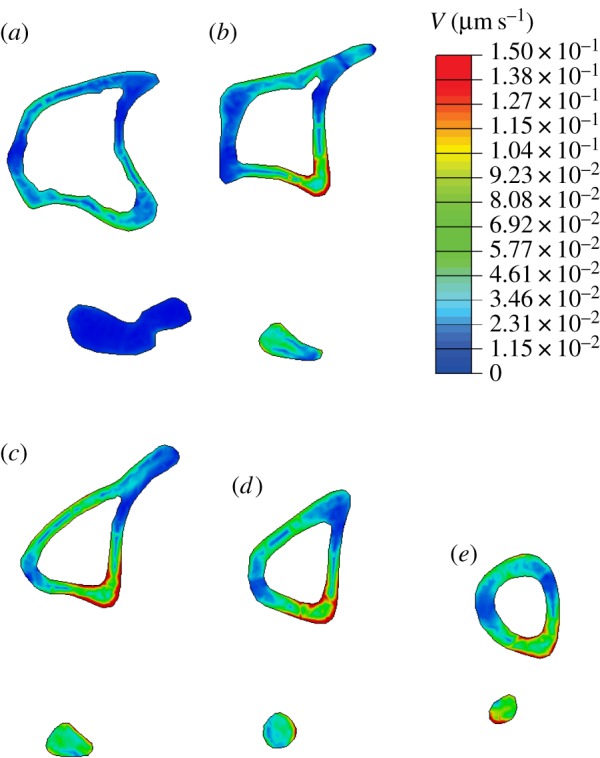
Contour plots of fluid velocity, *V*, obtained at *t* = 0.025 s (peak load) for different *Z* positions in the initial geometry. Higher fluid velocity magnitudes obtained in the medial surface and around the interosseous crest. (*a*) *Z* = 0.1, (*b*) *Z* = 0.3, (*c*) *Z* = 0.5, (*d*) *Z* = 0.7 and (*e*) *Z* = 0.9.

Figure 9 shows the contour plots obtained for fluid velocity magnitude at the time of peak load (*t* = 0.025 s) in the initial, unadapted geometry. Owing to the higher permeability of the membrane, the boundaries at the periosteum and endosteum develop low pore pressures, leading to higher fluid velocity magnitudes in the regions adjacent to the inner and outer surfaces.

Interestingly, the fluid in this model flows in the opposite direction to intuitive predictions; the interosseous crest region, which experiences compressive strains, develops low pore pressures during loading (influx of fluid) and high pore pressures during unloading (efflux of fluid). Conversely, the medial surface, in tension, develops pore pressures during loading, then low pore pressures during unloading. This counterintuitive flow direction is illustrated in the electronic supplementary material, figure S2, and is consistent with previous studies [32,37].

The time-varying profiles of fluid velocity were considered to be the mechanical stimulus used in the adaptation algorithm and the resulting experimental and simulated changes in bone morphology were compared using positional changes in cortical thickness.

### 3.3. *In vivo* adaptive response

Second moment of area measurements (electronic supplementary material, figure S1), calculated for all specimens, increased with statistical significance in 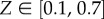 when the loaded limb was compared with the non-loaded limb. This provided evidence for the anabolic effect of mechanical loading.

**Figure 10.**
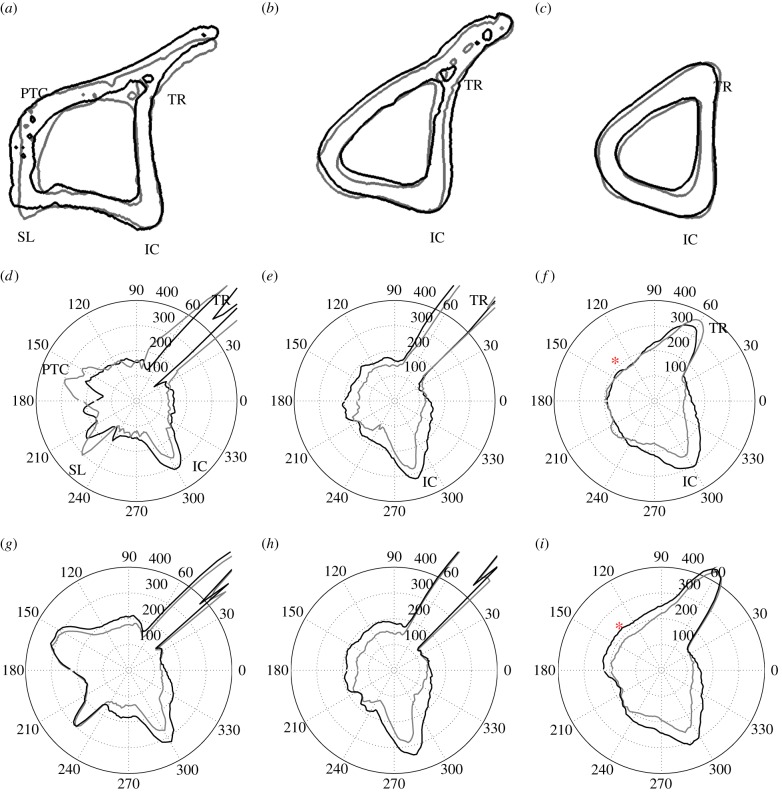
(*a*–*c*) Cross-sectional contours of left (non-loaded, grey line) and right (loaded, black line) tibiae, and respective polar representation cortical thickness, Th, for (*d*–*f*) the *in vivo* data measurements and (*g*–*i*) predictions using *Ψ*_A_ = 50 MPa. The red asterisk (*c* and *i*) is placed at *Z* = 0.7 and *θ* = 135° and highlights a region where *in silico* adaptation was overestimated (Th units in micrometres). (*a*) *Z* = 0.3, (*b*) *Z* = 0.5, (*c*) *Z* = 0.7; (*d*) micro-CT scan, *Z* = 0.3, (*e*) micro-CT scan, *Z* = 0.5, (*f*) micro-CT scan, *Z* = 0.7; (*g*) simulation, *Z* = 0.3, (*h*) simulation, *Z* = 0.5 and (*i*) simulation, *Z* = 0.7.

**Figure 11.**
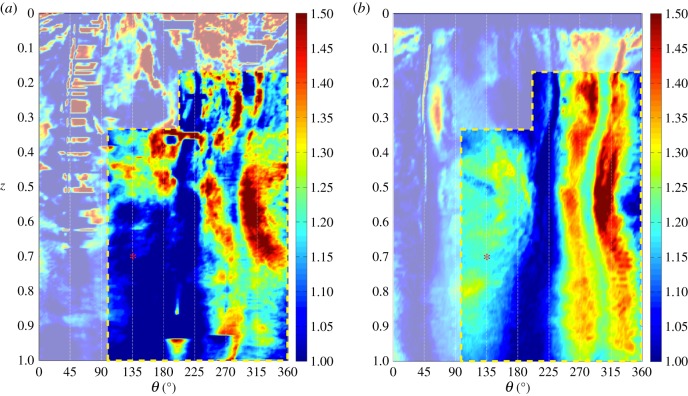
Colour map of changes in cortical thickness, ΔTh/Th, measured for *in vivo* data (*a*) against the respective predictions using *Ψ*_A_ = 50 MPa (*b*). The red asterisk is placed at *Z* = 0.7 and *θ* = 135°. Artefacts in the experimental data calculations predominate in the region masked over, outside the yellow dashed line. (*a*) micro-CT scan and (*b*) simulation.

Figure 10 shows cross sections of the specimen's tibia (*a*–*c*) and respective polar representations of cortical thickness, Th, calculated from micro-CT images (*d*–*f*) and from *in silico* simulations for the optimal value of *Ψ*_A_ (*g*–*i*), determined in §3.4. These are shown for control (grey line) and adapted (black line) tibiae, at *Z* = 0.3, 0.5 and 0.7. Figure 11 illustrates the calculations of changes in cortical thickness, ΔTh/Th, with colour maps plotted in a two-dimensional plane (*θ*, Z).

A common feature is the high thickness values calculated between *θ* = 40–60°, for *Z* < 0.7, a region which corresponds to the tibial ridge (TR) and peaks around 1000 µm. The interosseous crest (IC) is clearly notable as the local maxima around *θ* = 300° at all values of *Z*. The SL and the PC are better visible in figure 10, around *θ* = 220° and 160°, respectively.

Bone formation was observed in two regions, the medial surface (approximately *θ* = 90–180°, where maximum tensile strains were experienced) and the IC (approximately *θ* = 240–350°, in compression). Formation in the IC is present across the whole length of the diaphysis, whereas increase in bone thickness in the medial surface is most notable around the mid-diaphysis (figure 10*e*).

Some noise was present in the thickness measurements, particularly in proximal regions (figure 10*d*) and the TR. The TR region is observable in the colour map in figure 11*a* as the horizontal dashes of high ΔTh/Th values and occasional sudden longitudinal changes in thickness around *θ* = 50°. The main contributors for the presence of deceptive changes in thickness were (i) trabecular structures that, in some transverse sections, remained connected to the cortical shell (electronic supplementary material, figure S3), (ii) the opening and closing of foramina, both in the medullary cavity and in the periosteum (electronic supplementary material, figure S4), and (iii) small organ-level contralateral morphological differences that led to a misalignment of corresponding anatomical landmarks. These thickness artefacts were not considered as true adaptations (electronic supplementary material, figure S5).

Most of these features were contained outside the dashed box in figure 11*a*, i.e. the region that excludes the TR and the PC and the region with a higher concentration of connected trabecular bone, i.e. the first 15% of length. All regions where a reduction in cortical thickness was obtained were associated with calculation artefacts.

These results confirm that load-related increases in resorption were not found in the *in vivo* data. Cortical thickening calculations provided insights into the adaptive response and better understanding of the effects of external loading. We applied the same methodology to assess whether our computational and mechanobiological models matched these measurements.

### 3.4. Simulation of adaptation

Table 5 lists the iterations that matched the peak ΔTh/Th in the regions where bone formation occurred, for the considered values of *Ψ*_A_. Not surprisingly, with a lower threshold, fewer iterations were needed to reach a thickness change of 0.67.

**Table 5.** Final iteration obtained for each value of stimulus threshold, *Ψ*_A_.

*Ψ*_A_	iteration
1.0 × 10^−3^	7
3.0 × 10^−2^	13
6.0 × 10^−2^	12
7.0 × 10^−2^	13
1.0 × 10^−1^	15

Cortical thickness values obtained in this simulation are shown in polar coordinates in figure 10*g*–*i* and in a colour map in figure 11*b*. All simulations predicted load-related changes in architecture and increases in bone formation around the IC and in the medial surface, the regions of high mechanoadaptive response identified in the experimental data. Bone formation was overestimated in the distal half of the medial side (area comprised within *Z* > 0.55 and 90° < *θ* < 180°), as observable in the site marked by the red asterisk in figures 10*f*,*i* and 11. Increasing the value of the threshold *Ψ*_A_ resulted in a gradual confinement of adaptation to the regions where the measured strain and predicted mechanical stimulus *Ψ* were highest (i.e. the IC).

**Figure 12.**
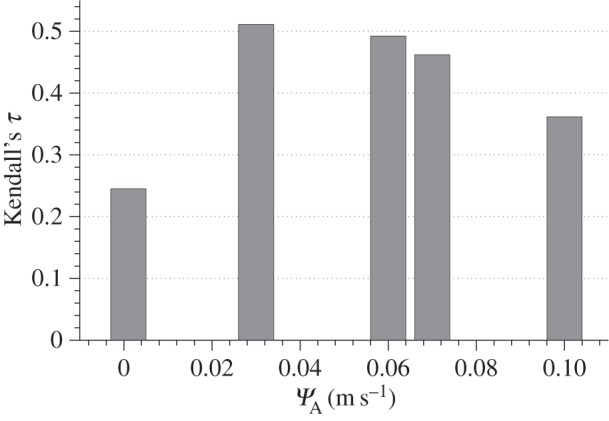
Kendall's *τ* rank correlation coefficient calculated between *in vivo* and *in silico* ΔTh/Th measurements obtained for the values of *Ψ*_A_ considered in this study. Simulations with an apposition threshold value of *Ψ*_A_ = 3 × 10^−2^ ms^−1^ obtained the strongest positive correlation (*p* < 0.001).

Figure 12 shows, for the different values of *Ψ*_A_, Kendall's *τ* value obtained when ΔTh/Th in 10 763 data points contained inside the dashed yellow lines in figure 11 (in order to exclude the areas with a high concentration of artefacts) was compared with the corresponding region in the simulation data. A value of *Ψ*_A_ = 3 × 10^−2^ ms^−1^ gave the best predictions (correlation coefficient *τ* = 0.51, *p* < 0.001) when compared with the *in vivo* response, showing a significant relatively strong positive correlation.

## 4. Discussion

The mouse tibial axial compression loading model is commonly used for studying load-induced anabolic effects in bone. We used FEA to determine the structural response of the tibia during mechanical stimulation and used poroelastic theory to estimate interstitial fluid flow. We propose a new mechanobiological formulation to predict cortical bone adaptation that results from non-static loading conditions. We determined the accuracy of our mechanobiological simulations compared with thickness changes quantified with micro-CT scans of loaded and non-loaded limbs of mice.

### 4.1. Cortical thickening measurements reveal sites of *in vivo* bone adaptation

Significant changes in cortical geometry were found between non-loaded and loaded bones, demonstrating the crucial role of mechanics in bone adaptation. The effects of expected daily habitual loading were not taken into consideration, as both legs were assumed to be exposed to similar physical forces during that regime. Previous studies found that surface peak strains, for 13 N applied loads, were up to 10 times higher than during normal walking and up to five times higher than during 13 cm jumps [23]. Despite the need for further studies that explore the relative contribution of normal cage activity versus extrinsic applied loading, these data provide strong evidence for the role of extrinsic mechanical loading as the major contributor for the adaptive response observed in the loaded leg.

The calculation of cortical thickness changes allowed for a comprehensive method for comparison of *in vivo* and *in silico* adaptation. Previous loading models in murine long bones reported ‘how much’ bone had formed in a specific cross section, using bulk cross-sectional parameters, but not ‘where’ it had formed in the cross section [23,26,38–40]. The method presented here provides spatial patterns of bone formation across the whole diaphysis that can be mapped to the distribution of strains, and other mechanical sequelae, that are engendered by the controlled application of load in this model. The capacity to control and modify these applied loads, together with the scope, shown herein, to map the distribution of the osteogenic response along the entire bone length, provides for added power in these analyses.

Our cortical thickness measurements showed that bone formation takes place predominantly on the medial surface and the IC, where our model, and experimental strain mapping with DIC, show high tensile and compressive strains. Some expected variability was seen on the location and extent of surface modelling in different samples, likely associated with the adaptation tolerance of each mouse, differential handling, loading of mice or physical activity outside loading times (fighting, cage climbing, etc.). Apart from one mouse, which did not exhibit any notable changes in cortical thickness in the whole diaphysis, all loaded tibiae had the highest amounts of bone formation in the IC, which matched histological regions found in single sections [23]. Some studies have shown woven bone response at very high loads using this model [41]; none of our mice demonstrated woven bone formation in the tibial shaft.

Measurement artefacts, in our loaded–non-loaded micro-CT comparisons, were easily identified, allowing focus on the true functional adaptation regions. Further developments, such as the removal of the effect of trabecular bone and contralateral misalignment, will improve this method. Non-rigid registration of each bone to the contralateral equivalent could be included, as done in previous human hip fracture studies, where regions of cortical thinning were determined [42]. This would allow statistical comparison of several mouse studies and facilitate the examination of thickness maps obtained from micro-CT scans. An alternative methodology would be time-lapsed imaging of bone morphometry [43], which combines *in vivo* micro-CT with registration of the same bone at different time points. Yet, the presented methodology, using *ex vivo* specimen imaging of contralateral bones, allows the morphology to be compared using images from conventional scanners, which are highly accessible.

### 4.2. Constitutive assumptions for permeability of bone and membranes

The correct order of magnitude of bone permeability is still a matter of debate [44]. Previous computational studies [20] showed that poroelastic models with bone permeability *k* = 10^−22^ m^2^ simulate relaxation times similar to *in vivo* measurements in mice and are consistent with recent experimental estimations [45].

The periosteal and endosteal permeabilities are commonly assumed to be, respectively, very low and very high, when compared with bone tissue. However, new experimental measurements raise questions regarding the assumption of an impermeable periosteum. Evans *et al*. [36] measured ovine periosteal permeability to be higher, of the order of 1 × 10^−17^ m^2^, for low flow rates, roughly 5 orders of magnitude higher than the permeability of the lacunar–canalicular porosity. The same value was assigned to both surfaces, despite the fact that the literature often characterizes the endosteum to be more permeable than the periosteum. However, owing to the considerable offset between lacunar–canalicular and periosteal permeability (5 orders of magnitude), poroelastic models were insensitive to any value of endosteal permeability above 1 × 10^−17^ m^2^.

### 4.3. Fluid velocity as a stimulus for bone adaptation

Darcy fluid velocity was considered as the *local* mechanical stimulus following recent observations that suggest that load-induced fluid flow is the likely mechanism for mechanocoupling [14–16]. Integrins [46], primary cilia [47] and stretch-activated ion channels [48] may be involved in transduction of the flow signal to the cell. Two mechanoreception mechanisms have been proposed to explain the role of fluid flow in mechanosignalling: (i) fluid shear stress acting on the cell membrane [14] and (ii) fluid drag force acting on the pericellular matrix fibres that tether cell transmembrane proteins to proteins in the extracellular matrix [49].^4^ According to Weinbaum *et al*. [16] and You *et al*. [50], the shear forces applied at the osteocyte process, *F*_s_, and the drag forces applied on the transverse pericellular fibres, *F*_d_, both depend on the geometry of canaliculi and cellular processes, the permeability of the extracellular space (dictated by the arrangement of fibres) and the spatial gradient of fluid pressure along the direction of the canaliculi, *∂p*/*∂y*. In our poroelastic model, both the effect of the structural properties of the canaliculi and the intrinsic permeability at the macroscopic level, *k*, are considered to be homogenized at the continuum level. Therefore, the term *∂p*/*∂y* is the only one expected to vary in time and across the bone geometry. Under these assumptions, *F*_s_ and *F*_d_ are proportional to each other [50] and proportional to the apparent fluid velocity, *V*,4.1
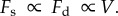


Hence, fluid velocity provides, at the continuum level, a good approximation to both shear and drag forces and should be a suitable mechanical stimulus to simulate adaptation in computational studies.

### 4.4. Mechanobiological model captured the *in vivo* bone formation patterns

Our mathematical model was able to accurately predict the areas of adaptation and reproduce the distributions of load-induced cortical thickening observed *in vivo*. The statistically significant and relatively strong correlation of our simulations with the measured data suggest that our model is suitable to predict cortical bone adaptation in the murine loading model and that poroelastic models can be used to understand a possible link between fluid flow and loading parameters, at the organ level. The simulations were most accurate in the mid-diaphysis and less accurate proximally, due to the artefacts in the experimental measurements. There were also small discrepancies in the distal half of the medial side as indicated in figures 10 and 11, likely due to inevitable simplifications in our approach, such as homogeneous constitutive and mechanobiological assumptions (e.g. threshold stimuli and osteocyte distribution), loading conditions and implicit modelling of biochemical processes.

The presented mechanobiological model is proposed as a time-dependent analogy to the trilinear curve referred to in §2.3. Trilinear stimulus–adaptation functions are commonly used in bone adaptation studies [5–7,10,17,28], based on various studies showing a dose-dependent relationship between higher mechanical stimulation and bone formation response [51–53]. In this study, we adjusted the stimulus–adaptation relationship, exploring different slopes and thresholds for bone formation, leading to a step-function profile that suggests a discrete instantaneous response from bone cells. This does not necessarily mean that osteoblasts are not capable of proportionally adjusting their response to mechanical signals. One could hypothesize that bone cells integrate discrete signals over time that result in a continuous tissue response.

The mechanical stimulus threshold for bone accretion, *Ψ*_A_ (and, if modelled, the threshold for resorption, *Ψ*_R_), dictates the spatial extent of the adaptive region. The lower its value, the more sites will respond to the loading, and thus the larger this region will be. Regions exposed for longer periods to mechanical stimulation will build up higher amounts of formed tissue, integrating non-static load conditions. As a result, *Ψ*_A_ also regulates the amount of added bone. Biologically, both threshold values *Ψ*_A_ and *Ψ*_R_ are likely to be regulated by multiple factors, such as the cellular network, metabolic and nutrient availability, or the local material properties of bone, and may not be constant within bone tissue.

Using a fluid-related stimulus captures the influence of the time dependence of adaptation, specifically the effects of load rate, frequency [17–19] and rest periods between load cycles [20,21]. Strain and stress, used as mechanical stimuli for adaptation, are insensitive to these parameters. Yet fluid flow as a mechanical stimulus is not capable of modelling long-term saturation in the functional adaptation of bone mass for an increasing number of load cycles per day [54] or the recovery of mechanosensitivity in protocols that include rest-insertion periods of the order of hours [55] or days [56]. Future work will extend this, by examining how cells desensitize following persistent mechanostimulation, in the longer term. In addition, further developments of this study will look at how the presented adaptation law translates to different animal models, ages and load parameters.

This is the first time, to our knowledge, that a cortical bone adaptation model has been accurately predicted with three-dimensional spatial resolution. The ability to locally predict where bone will form under a given mechanical load has implications for optimizing non-pharmacological therapies that direct bone adaptation with mechanical loads. This can potentially contribute to the integration of poroelastic formulations in a multi-scale framework of load-induced bone adaptation. This model will use this capability in future studies to optimize the loading regime. The shift from postdictive to predictive models will empower researchers to understand where and how much bone will form under defined loading conditions, which eventually will enable clinicians in the design of optimized external loads or exercise regimes that are able to address bone deficits in clinical diagnoses linked to bone loss.

## Supplementary Material

Supplementary Figures S1-S5

## Endnotes

1 As shown in the electronic supplementary material, figure S1*a*, the FE model indicated that tibial loading induced bending around an axis very similar to the geometric minor axis. Therefore, adaptive changes are more likely to be measured around the minor axis.

2 Interestingly, both average (≈150 µm) and maximum (≈350 µm) values of cortical thickness in mice (apart from the tibial ridge regions) had similar values to those observed in osteon radius [30]. Mouse bones do not have osteons, which may limit their physiologically allowed thicknesses.

3 The Poisson ratio value used in this study is more appropriate for poroelastic cartilage. However, a study was conducted that showed that considering an incompressible growth plate, with *v*_t_ = *v*_lt_ = 0.49, leads to minor changes in the mechanical environment of the diaphysis.

4 Drag forces on the pericellular matrix were estimated to be 20 times higher than shear forces [50], suggesting that drag forces may be the most likely mechanosignalling mechanism.

## Data accessibility

Data deposited in the GitHub repository: https://github.com/afpereira/mouseTibiaData.
