# Supplementary material for: Predicting cortical bone adaptation to axial loading in the mouse tibia
Source: J R Soc Interface. 2015 Sep 6;12(110):20150590. doi: 10.1098/rsif.2015.0590 (PMC4614470; doi:10.1098/rsif.2015.0590)
Supplement: Supplementary Figures S1-S5 [file rsif20150590supp1.pdf]

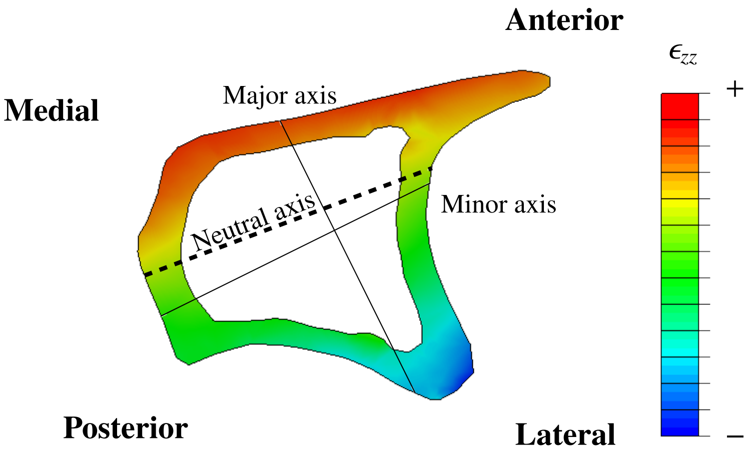

(a)

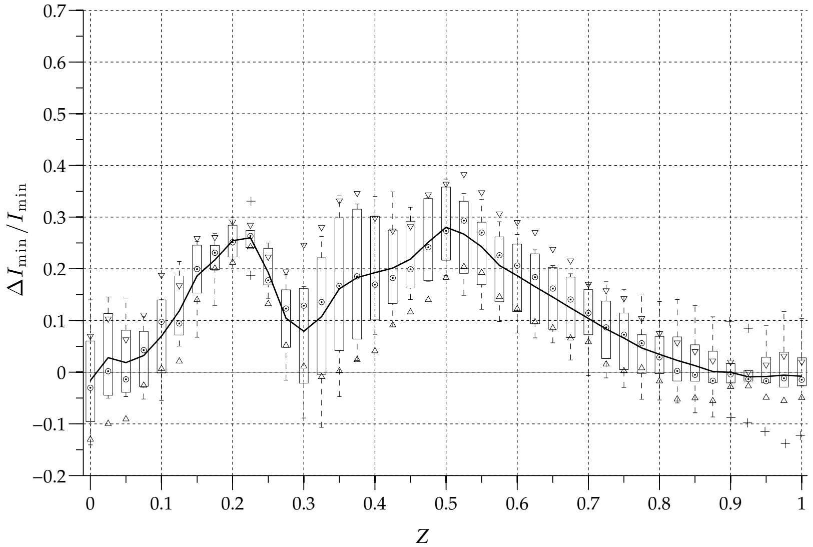

(b)

Figure S1: Second moment of area about the minor axis. (**a**) Colormap of the longitudinal strains in a diaphyseal cross-section, calculated with a finite-element model of the bone, with the second moment of area major and minor axis (solid lines) and zero-strain neutral axis (dashed line). Tensile (positively strained) and compressive (negatively strained) sites are show with warm and cold colours respectively. Bone adaptation is expected where mechanical signal is highest, away from the neutral axis, which is close to the minor axis. Hence $I_{\min}$ is used to capture changes in cortical shape. (**b**) Boxplot representation of tibial changes in second moment of area about the minor axis, as a function of the normalised diaphyseal length Z. A statistically significant increase in second moment of area was indicated when the confidence intervals did not contain 0.

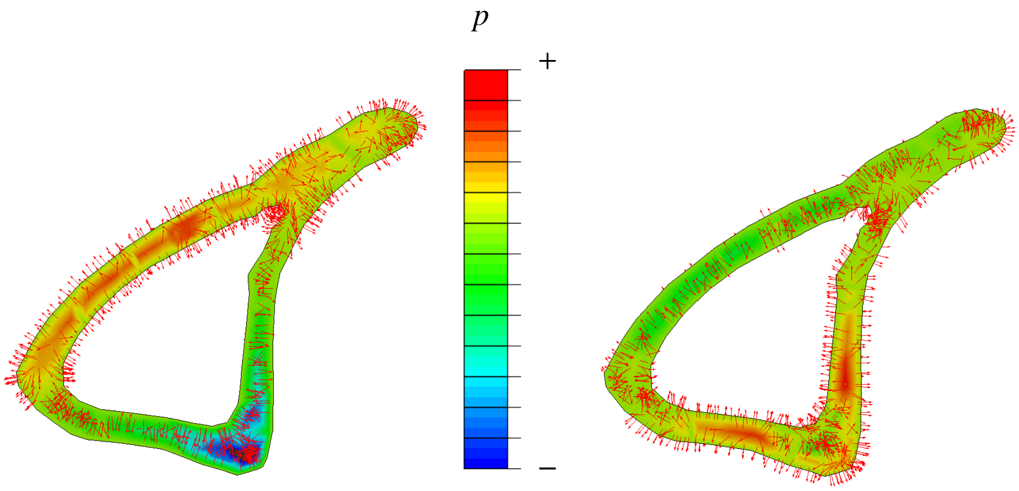

(a) Loading

(b) Unloading

Figure S2: Vector field visualisation of peak interstitial fluid velocity during load (**a**) and unload (**b**). Cross sections at $Z = 0.5$ with contour plots of pore pressure, p, and unit length fluid flow vectors represented by the red arrows (right). Regions in compression experience an influx of fluid during loading, while the reverse happens in regions in tension.

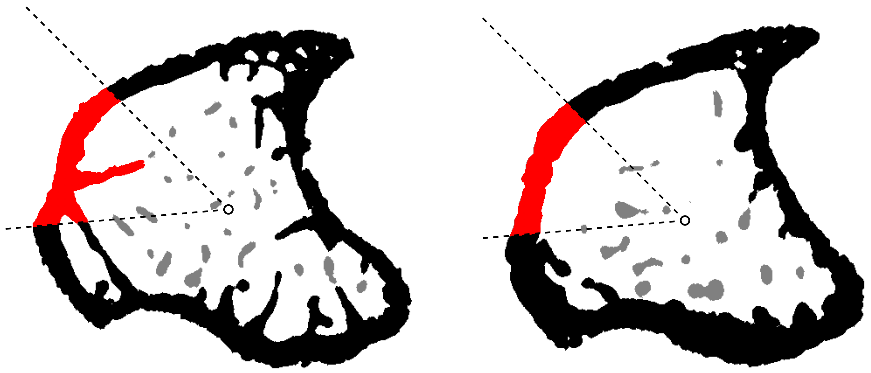

(a) Left (Control)   (b) Right (Loaded)

Figure S3: Left and right tibiae of the specimen at $Z = 0.045$ and angle range of $135° < \theta < 185°$ highlighted in red. Trabecular structures are present in the left side, leading to erroneous calculations of the thickness for this limb.

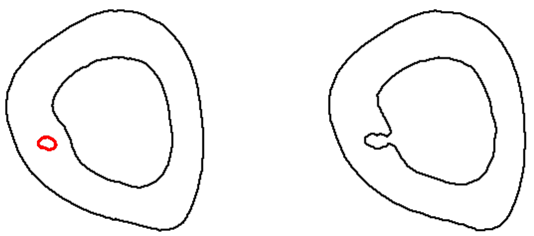

(a) $Z = 0.93$ (b) $Z = 0.94$

Figure S4: Opening of blood vessel in the endosteal boundary of the left tibia. When the vessel is enclosed intracortically at a certain section (highlight with red in a) it is not taken into consideration for the calculation of cortical thickness. The opening cause sudden changes in cortical thickness readings.

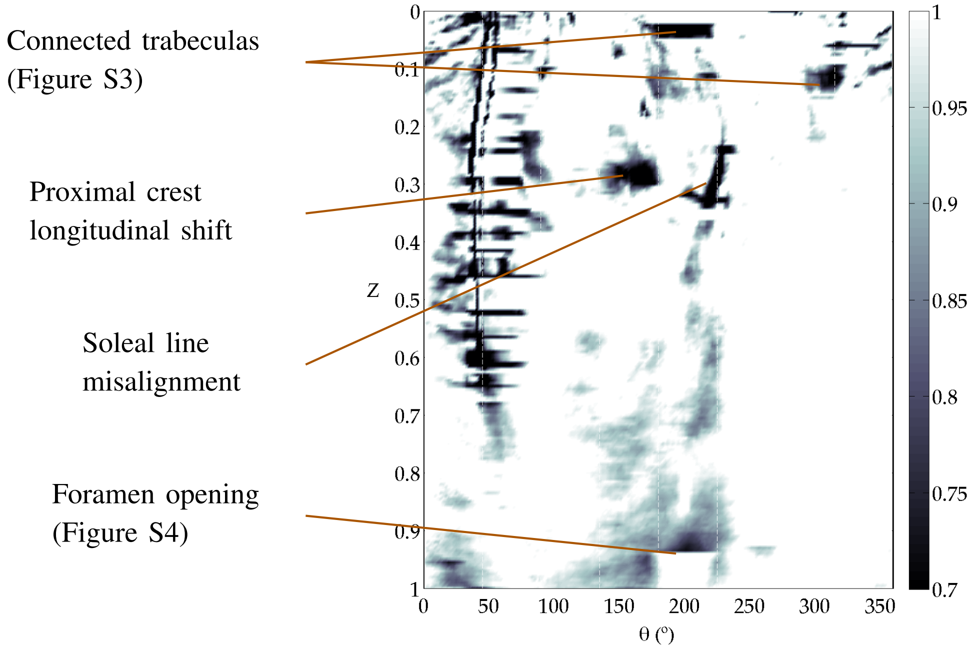

Figure S5: Thickness map of predicted resorptive areas in dark shades ($0.7 < \Delta Th/Th < 1$). Many of these regions, as pointed in the figure, are in fact artefacts that result from misalignment of bony landmarks between sides.
